# A Randomized Controlled Trial Comparing Methotrexate Iontophoresis and Oral Methotrexate for the Treatment of Hyperkeratotic Palmoplantar Eczema

**DOI:** 10.7759/cureus.83684

**Published:** 2025-05-07

**Authors:** Suvigya Sachan, Mitanjali Sethy, Shibashis Chatterjee, Laxman Besra, Satyajit Sahu, Hemanta K Kar

**Affiliations:** 1 Department of Dermatology, Venereology, and Leprosy, All India Institute of Medical Sciences, Mangalagiri, IND; 2 Department of Dermatology, Venereology, and Leprosy, Kalinga Institute of Medical Sciences, Bhubaneswar, IND; 3 Department of Dermatology, Venereology, and Leprosy, Shri Ramkrishna Institute of Medical Sciences and Sanaka Hospitals, Durgapur, IND; 4 Department of Dermatology, Ispat General Hospital, Rourkela, IND

**Keywords:** chronic eczema, hand foot eczema, hyperkeratotic palmoplantar eczema, methotrexate iontophoresis, oral methotrexate

## Abstract

Background: Palmoplantar hyperkeratotic eczema is quite disabling and affects a person’s daily quality of life. Among the various treatment options available, oral methotrexate was quite effective. However, it was associated with systemic and cutaneous side effects. Therefore, delivery of methotrexate through Iontophoresis was an option to reduce the adverse effects and to enhance the absorption and efficacy.

Objectives: To observe the efficacy and the adverse effects of methotrexate iontophoresis as compared to oral methotrexate therapy in the treatment of hyperkeratotic palmoplantar eczema.

Methodology: This unblinded, randomized, parallel-group controlled trial included 88 patients. Group A (*n *= 44, 50%) received weekly iontophoresis of injectable methotrexate for four weeks, then biweekly for up to three months. Group B (*n* = 44, 50%) received oral methotrexate (7.5 mg/week) with gradual dose increases. Efficacy was assessed using Hand Eczema Severity Index (HECSI) scores, digital photography, and follow-up evaluations conducted three months after study completion.

Results: Both groups showed significant improvement in HECSI scores (*P* < 0.05) for palms and soles, with no statistical difference in percentage improvement. Group A reported cutaneous side effects like blistering and itching, while Group B had systemic side effects, including headache, nausea, and elevated liver enzymes. Relapse rates were 9.1% (*n* = 4) in Group A and 2.27% (*n* = 1) in Group B.

Conclusions: Iontophoretic delivery of methotrexate in palmoplantar hyperkeratotic eczema was equally effective as oral methotrexate.

## Introduction

Palmoplantar eczema ranks among the most common dermatological diseases [1]. Studies indicate that approximately 2%-10% of the population will likely develop this condition during their lifetime [2]. As one variant of palmoplantar eczema, hyperkeratotic palmoplantar eczema manifests with moderate to severe pruritus, characterized by thick, scaly, fissured, infiltrated lesions on the palms, soles, fingers, and toes. Despite its prevalence, the exact etiology of this condition remains unestablished [3].

The debilitating nature of palmoplantar hyperkeratotic eczema cannot be overstated. Many patients struggle with performing routine activities, and in severe cases, some individuals are even forced to change their occupations due to the functional limitations imposed by this disorder [4]. Currently, numerous treatment options exist, including topical corticosteroids, calcineurin inhibitors, coal tar, emollients, ultraviolet therapy, and topical ruxolinitib, producing varying results. For more severe cases, systemic treatments such as oral corticosteroids and immunosuppressive agents (methotrexate, cyclosporine, and azathioprine) are available; however, the associated side effects remain a significant concern for both patients and physicians alike [3]. The off-label use of biologics such as dupilumab, tralokinumab, and lebrikizumab has shown promising results in a limited number of reported hand eczema cases. Additionally, the potent topical pan-JAK inhibitor, delgocitinib, is anticipated to receive approval soon.

Among the systemic medications, methotrexate stands out as both affordable and relatively effective. Nevertheless, its use is associated with potentially serious hematological, hepatic, pulmonary, gastrological, and mucocutaneous side effects [5]. To mitigate these adverse reactions, researchers have experimented with topical applications of methotrexate. Unfortunately, results with conventional topical methotrexate have been inconsistent, primarily due to poor drug penetration through the skin barrier. In response to this challenge, the delivery of methotrexate through iontophoresis has emerged as a promising alternative that may reduce adverse effects while enhancing both absorption and efficacy [4].

Iontophoresis represents a non-invasive technique that enhances drug penetration into the skin through the application of an electric current [6]. By creating an ion-electric field, this method drives ions through the skin while simultaneously increasing dermal permeability through electrical current. As a localized treatment approach, iontophoresis significantly reduces the risk of systemic side effects and avoids potential secondary diseases often associated with systemic therapeutic modalities [6]. Therefore, we undertook this study to evaluate the efficacy and adverse effects of methotrexate iontophoresis compared to conventional oral methotrexate therapy in the treatment of palmoplantar hyperkeratotic eczema.

This article was previously presented as a meeting abstract at the 25th World Congress of Dermatology, held in Singapore on July 5, 2023.

## Materials and methods

Patients with palmoplantar hyperkeratotic eczema who attended the outpatient department of dermatology, venereology, and leprosy from October 2018 to August 2020 were investigated in this randomized, unblinded, parallel-group controlled trial.

Ethical considerations

The Institutional Research and Ethical Committee approved the study (Ref. KIMS/KIIT/IEC/120/2018, dated: 07.09.2018). The study was registered with the Clinical Trials Registry - India (CTRI) under the trial number CTRI/2019/08/020770.

Sample size

The required sample size was calculated to be 88 (44 for each group) based on an assumed standard deviation of 1.0, expected difference of 0.60, 5% significance level, and 80% power. To account for potential dropouts, an additional 10 cases were included in the study.

Consort diagram

**Figure 1:**
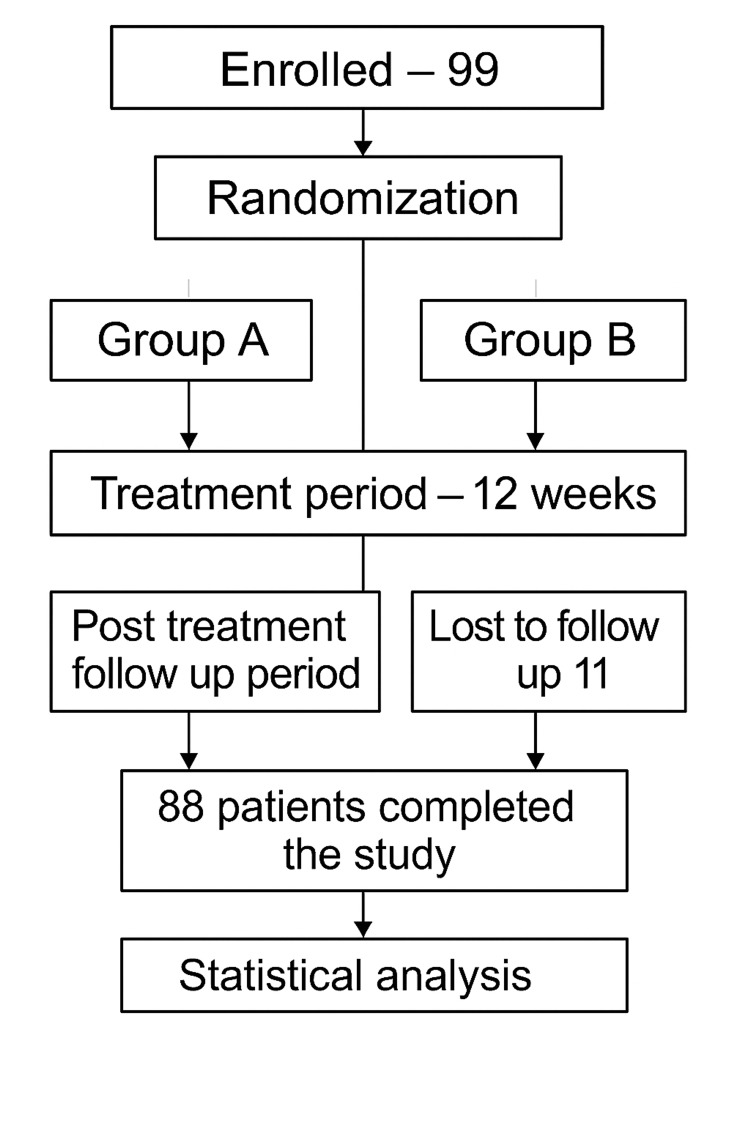
The CONSORT diagram outlining the flow of participants throughout the study. CONSORT, Consolidated Standards of Reporting Trials

Figure 1 shows the Consolidated Standards of Reporting Trials (CONSORT) diagram.

Inclusion and exclusion criteria

The study included patients between 18 and 60 years of age who had been diagnosed with palmoplantar hyperkeratotic eczema persisting for more than eight weeks. "However, several categories of patients were excluded, including those unwilling to undergo follow-up; pregnant or lactating women; individuals with renal, hepatic, or hematological disorders; patients with malignancies; those with pacemakers, cardiac arrhythmias, or metallic implants; patients with known hypersensitivity to methotrexate; those currently receiving other immunosuppressive agents; and individuals who had used systemic or topical steroids for more than two months.

A total of 99 patients were enrolled in the study and randomized into two treatment groups using a concealment method involving opaque envelopes. Group A, the intervention group, received methotrexate iontophoresis, while Group B, the comparator group, was treated with oral methotrexate. During the study, 11 patients withdrew: six from Group A and five from Group B.

During the initial visit, a comprehensive medical history was obtained and a thorough physical examination was conducted for each participant. The diagnosis of eczema was established based on several factors: patient history, presenting symptoms, duration of the condition, local examination findings, and dermoscopic evaluation. Hyperkeratotic palmoplantar eczema was diagnosed based on the clinical features used to identify hyperkeratotic variants, such as marked thickening of palmoplantar skin, scaling, fissuring, and hyperkeratotic plaques. Skin biopsy was performed for histopathological confirmation in cases where the diagnosis was uncertain, which was necessary in 42% of patients with ambiguous presentations. All participants provided informed written consent before enrollment.

Before beginning treatment, baseline investigations were conducted, including complete blood count, liver and renal function tests, and urine analysis. These tests were subsequently repeated monthly throughout the six-month study period (which included a three-month follow-up phase). Hand Eczema Severity Index (HECSI) scores were recorded before and after treatment, and photographs were taken at regular intervals to support objective clinical assessment.

Treatment modality

Group A

Iontophoresis was administered to palms or soles using an injectable preparation of methotrexate (25 mg/mL). The treatment schedule consisted of weekly sessions for the first four weeks, followed by biweekly applications until a response was observed, continuing for a maximum of three months. Following the treatment phase, patients were monitored monthly for three consecutive months to assess both improvement and potential side effects.

The methotrexate dilution process involved mixing 25 mg of 1 mL of methotrexate in 24 mL of sterile water, resulting in a final concentration of approximately 1 mg/mL (1:1 ratio). A gauze pad was saturated in this solution and placed on the affected palm or sole. Additionally, the dorsal aspect of the hand/foot was covered with a wet gauze pad. Both the palm/sole and dorsal aspects were then wrapped in aluminum foil, with the treated extremity positioned on the cathode end of the iontophoretic plate. To complete the circuit, the untreated hand/foot was similarly prepared with wet gauze and aluminum foil and then placed on the anode end of the plate. Water-filled trays on both sides allowed for complete immersion of the hands/feet.

**Figure 2:**
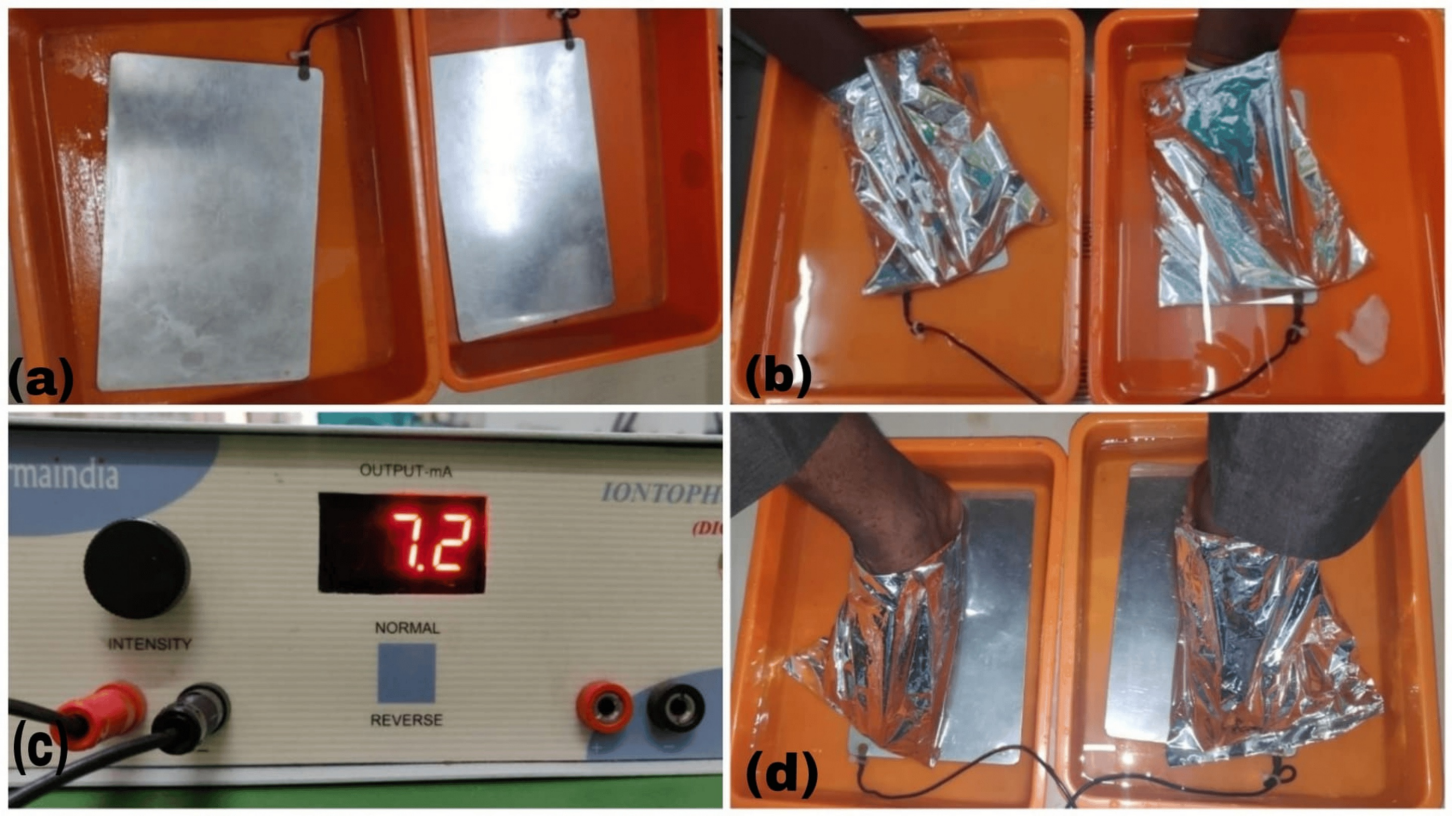
(a) Iontophoresis machine with electrode plates; (b) methotrexate delivery to the palms of a patient; (c) iontophoresis machine; (d) methotrexate delivery to the soles of a patient.

Once prepared, the iontophoresis process began with direct current passing through the solution, facilitating methotrexate delivery to affected areas. Patients typically experienced a mild tingling sensation as the current penetrated the skin. The current strength was maintained between 5 and 10 mA for 8-10 minutes, and adjusted according to each patient's tolerance level. Notably, most patients comfortably tolerated the current in the range of 7 to 8 mA, as demonstrated in Figures 2a-2d.

When there was involvement of both palms and soles in a single session, first iontophoresis was done on the palms, then on the soles in the same session.

Group B

Treatment with oral methotrexate began with a 5 mg test dose, followed by 7.5 mg weekly. The dosage was then gradually increased by 2.5 mg weekly to a maximum of 15 mg weekly until a therapeutic response was observed, or three months had elapsed.

For both groups, patients underwent monthly follow-up examinations for three months to monitor the clinical improvement of lesions and to document any treatment-related side effects.

Outcome measures

The study assessed multiple outcome parameters: treatment efficacy (determined by comparing pre- and post-treatment HECSI scores), improvement of lesions following treatment, adverse effects, relapse incidents during the follow-up period, and adverse effects observed during follow-up.

Statistical analysis

All collected data were organized and tabulated using Microsoft Excel. Results for continuous data were expressed as mean ± standard deviation, while categorical data were presented as frequencies and cross-tables. To evaluate significant changes in HECSI scores for both palm and sole after treatment, paired t-tests were utilized. Independent t-tests were employed to assess significant differences in percentage improvement between palm and sole measurements. All statistical analyses were performed using SPSS, version 20.0 (IBM Corp., Armonk, NY), with a *P*-value < 0.05 considered statistically significant.

## Results

Table 1 presents the baseline characteristics of both treatment groups. 

**Table 1:** Baseline characteristics of both groups.

	Group A	Group B
Mean age	43.3 ± 10.8 years	44.0 ± 11.9 years
Male, *n* (%)	26 (59.1%)	21 (47.7%)
Female, *n* (%)	18 (40.9%)	23 (52.3%)
Male:female ratio	1.4:1	0.9:1
Duration of symptoms	11.4 ± 6.5 months	13.6 ± 9.6 months
Symptoms		
Itching, *n* (%)	26 (59.1%)	32 (72.7%)
Fissuring, *n* (%)	19 (43.2%)	18 (40.9%)
Pain, *n* (%)	18 (40.9%)	11(25%)
Thickening, *n* (%)	16 (36.4%)	2 (45.5%)
Hyperpigmentation, *n* (%)	12 (27.3%)	5 (11.4%)
More than one symptom, *n* (%)	38 (86.36%)	30 (68.18%)
Occupation		
Farmer, *n* (%)	10 (22.77%)	6 (13.60%)
Housewife, *n* (%)	14 (31.80%)	20 (45.50%)
Office worker, *n* (%)	8 (18.20%)	5 (11.40%)

Demographic analysis reveals comparable age distributions between the groups, with a mean age of 43.3 years in Group A and 44 years in Group B. However, gender distribution showed some variation, with Group A comprising a higher proportion of males (26, 59.1%), while Group B included more females (23, 52.3%). Additionally, patients in Group B reported a slightly longer duration of symptoms compared to those in Group A.

Regarding symptomatology, itching emerged as the predominant complaint across both groups, though its prevalence was noticeably higher in Group B (32, 72.7%) compared to Group A (26, 59.1%). Other clinical manifestations such as fissuring, pain, and thickening exhibited comparable frequencies between the two groups. Interestingly, hyperpigmentation was observed more frequently among patients in Group A. When analyzing symptom complexity, a greater percentage of patients in Group A (38, 86.4%) presented with multiple symptoms simultaneously, compared to Group B (30, 68.2%).

Occupational distribution patterns also differed between the groups. Group B featured housewives as the largest occupational category, whereas Group A demonstrated a more balanced distribution across various professional categories.

**Figure 3:**
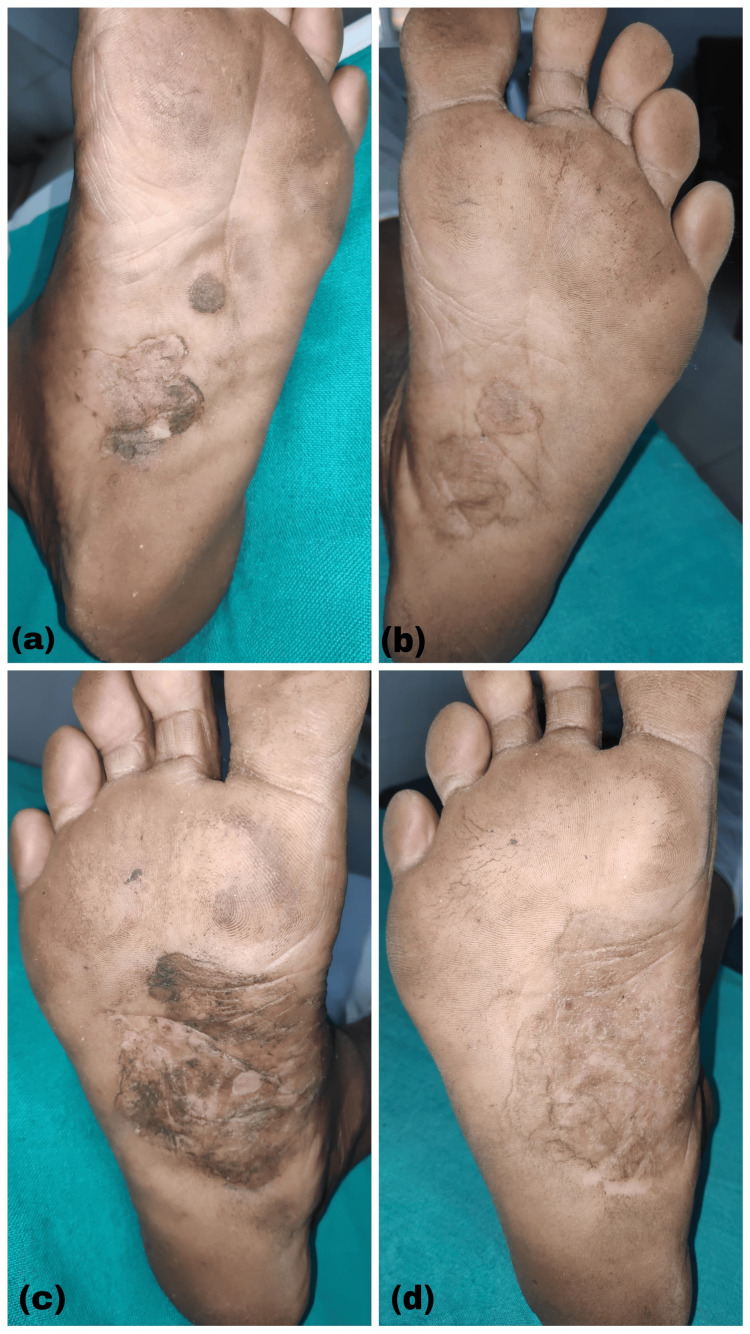
(a, c) Lesions before treatment; (b, d) lesions after 12 weeks of treatment in Group A.

**Figure 4:**
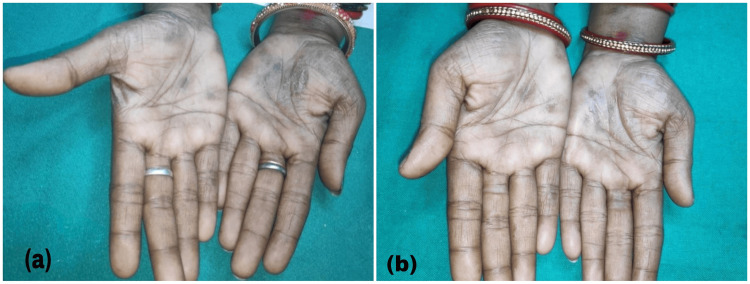
(a) Lesions before treatment; (b) lesions after 12 weeks of treatment in Group B.

Clinical improvement was documented through a comprehensive photographic assessment. Pre- and post-treatment photographs of palms and soles were meticulously compared, as illustrated in Figures 3a-3d and Figures 4a-4b.

These visual comparisons provide compelling evidence of the therapeutic efficacy observed in both treatment modalities.

Efficacy

The therapeutic efficacy of both treatment modalities was rigorously evaluated through HECSI scores and subsequently analyzed using paired sample t-tests.

In Group A, significant reductions in HECSI scores were observed across multiple anatomical sites - palms, soles, and the combined palm-sole regions. Group B demonstrated similarly impressive decreases in severity scores following treatment intervention. Statistical analysis revealed that the pre-and post-treatment changes in HECSI scores were highly significant in both treatment groups (*P* < 0.05), confirming the clinical effectiveness of both therapeutic approaches. Tables 2-3 provide comprehensive details of these statistical outcomes.

**Table 2:** Hand Eczema Severity Index scores (HECSI) of Group A before and after treatment. A paired sample t-test was used for analysis, with *P* < 0.05 considered statistically significant. Effect sizes are reported as Cohen’s *d*.

HECSI score		Number of patients	Mean	Std. deviation	95% confidence interval of difference	Cohen’s d	t-value	*P*-value
Palms	Before treatment	11 (100%)	7.36	4.63	2.10-7.34	1.19	3.96	0.002
	After treatment	11 (100%)	2.63	3.90				
Soles	Before treatment	12 (100%)	15.66	11.78	5.53-14.29	1.39	4.83	0.000
	After treatment	12 (100%)	5.75	5.72				
Both palms and soles	Before treatment	21 (100%)	21.90	13.89	8.70-18.06	1.29	5.89	0.001
	After treatment	21 (100%)	8.52	5.61				

**Table 3:** Hand Eczema Severity Index scores (HECSI) of Group B before and after treatment. A paired sample t-test was used for analysis, with *P* < 0.05 considered statistically significant. Effect sizes are reported as Cohen’s *d*.

HECSI score		Number of patients	Mean	Std. deviation	95% confidence interval of difference	Cohen’s *d*	t-value	*P*- value
Palms	Before treatment	10 (100%)	10.3	7.51	3.38-9.41	0.97	4.67	0.001
	After treatment	10 (100%)	3.9	5.34				
Soles	Before treatment	17 (100%)	18.05	12.90	6.38-16.79	1.13	4.67	0.002
	After treatment	17 (100%)	6.47	5.64				
Both palms and soles	Before treatment	17 (100%)	33.88	32.81	9.31-27.16	0.67	4.27	0.000
	After treatment	17 (100%)	15.65	17.54				

Comparative analysis of improvement patterns between the two groups revealed interesting nuances in treatment response. While both groups exhibited comparable overall improvement trends, certain site-specific variations were observed. Group A demonstrated a superior improvement in combined lesions affecting both palms and soles simultaneously. In contrast, Group B showed marginally greater improvement in isolated palm lesions. These subtle differences in treatment response patterns, although not reaching statistical significance, suggest potential variations in the distribution and bioavailability of methotrexate depending on the route of administration.

Percentage Improvement of Lesions

As shown in Table 4, both treatment groups demonstrated similar percentage improvements in lesions affecting the palms and soles. 

**Table 4:** Comparison of percentage improvement of lesions in the palms and soles in both groups. An independent sample t-test was used for statistical analysis, with a *P*-value < 0.05 considered statistically significant.

	Intervention	Mean (%)	Std. deviation	*P*-value
Improvement palm (%)	Group A	60.15	25.02	0.544
	Group B	64.31	27.37	
Improvement sole (%)	Group A	60.21	23.06	0.959
	Group B	60.48	20.31	

Group B exhibited a marginally higher mean improvement in palm lesions compared to Group A; however, this difference did not reach statistical significance. A comparable pattern emerged in the treatment of sole lesions, with both therapeutic approaches achieving approximately 60% clinical improvement. These findings suggest equivalent efficacy between methotrexate iontophoresis and oral methotrexate administration. Statistical validation through independent t-test analysis confirmed the absence of significant differences between the two treatment modalities (*P* > 0.05). Figures 3, 4 provide visual documentation of the clinical transformation in representative cases from Groups A and B, illustrating the pre-and post-treatment appearance of lesions.

Adverse effects

Adverse Effects Observed in Group A

The adverse effect profile in Group A was predominantly characterized by localized cutaneous reactions. Among the 44 patients receiving methotrexate iontophoresis, one patient (2.27%) developed blisters over the dorsal aspect of both hands and feet following treatment. Post-inflammatory hyperpigmentation was observed in two patients (4.54%), while two others (4.54%) reported sensations of electric current during the administration of therapy. Additionally, one patient experienced severe itching over the treatment area after the initial iontophoresis session, necessitating discontinuation of therapy. Notably, no patients in this group reported systemic adverse effects.

Adverse Effects Observed in Group B

In contrast, Group B patients primarily experienced systemic adverse reactions. Of the 44 patients receiving oral methotrexate, two (4.5%) reported headaches, four (9.1%) experienced nausea, and two (4.54%) developed episodes of vomiting. Laboratory abnormalities were also documented, with two patients (4.5%) showing mild elevations in liver enzymes. More concerning, two additional patients demonstrated greater than twofold increases in hepatic enzyme levels, requiring cessation of treatment. As expected, no cutaneous adverse effects were reported in this treatment group.

Relapse during follow-up

Concerning disease recurrence, four patients in Group A experienced relapse after completing the treatment regimen, with relapses occurring during the second and third months of the follow-up period. In comparison, only one patient in Group B experienced a relapse, which occurred during the third month of follow-up. Despite this numerical difference, statistical analysis revealed no significant variation in relapse rates between the two treatment groups (*P* < 0.05).

## Discussion

Several recent studies investigating methotrexate iontophoresis have demonstrated promising outcomes. Research by Haseena et al. [4] and Chandrappa et al. [7] revealed good response rates (>50%) with minimal side effects in patients with palmoplantar psoriasis. Specifically, the study conducted by Haseena et al. [4] documented significant improvement - 63.25% ± 22.70% in palms and 64.08% ± 19.96% in soles - after just eight weeks of methotrexate iontophoresis treatment. Similarly, Chandrappa et al. [7] reported satisfactory improvement (>50%) following six weeks of therapy. Although they noted burn injuries in 12 out of 25 patients, these injuries were generally trivial and self-limiting. Interestingly, Haseena et al. [4] did not report any adverse effects in their study. These encouraging findings motivated us to investigate methotrexate iontophoresis specifically for palmoplantar hyperkeratotic eczema and to determine whether its side-effect profile resembles that of oral methotrexate.

Our study results demonstrated considerable improvement in palmoplantar hyperkeratotic eczema across both treatment groups. However, we found no statistically significant difference between the two therapeutic approaches. This suggests that iontophoretic delivery of methotrexate for palmoplantar hyperkeratotic eczema is equally effective as conventional oral methotrexate administration.

The mean age of participants in our study population was 43 years, which aligns with findings from a study by Hersle et al. [8], who reported a mean age of 46 years. Furthermore, Veien et al. [9] observed that the majority of hand eczema cases occurred in the 20-40 year age range. Together, these observations indicate a predominance of hand and foot eczema in middle-aged individuals.

Regarding gender distribution, our study revealed a slight preponderance of males (47, 53.4%) compared to females (41, 46.6%). While many previous studies have reported hand eczema to be more prevalent among females [10], Nethercott et al. [11] documented findings similar to ours. This male predominance may be attributed to occupational factors - men typically work outside and thus experience greater exposure to chemicals and environmental triggers that contribute to palmoplantar eczema development. Additionally, health-seeking behavior tends to be more common among males than females in our study population.

In terms of anatomical involvement, our current study found that 21 patients (23.86%) exhibited palmar involvement exclusively, 29 (33%) showed plantar involvement, and 38 (43.18%) demonstrated both palmar and plantar involvement. By comparison, Sanjeev et al. [12] reported different distribution patterns in chronic dermatitis: 65% with palmar involvement, 7.9% with plantar involvement, and 27% with palmoplantar involvement. Disease duration in our study ranged from 2 to 36 months (mean duration 12.5 months), whereas Vishwender et al. [13], in their Rajasthan-based study on hand eczema, observed disease durations spanning from 6 to 60 months.

Occupation-wise, our study population comprised 34 housewives (38.6%), followed by 16 farmers (18.2%) and 13 office workers (14.8%). The prevalence among housewives likely stems from routine activities such as fruit and vegetable cutting and frequent exposure to soaps and detergents (wet work). Farmers developed palmoplantar eczema due to occupational hazards, particularly repeated exposure to insecticides and pesticides. Office workers, meanwhile, faced greater exposure to workplace allergens. These occupational patterns closely mirror findings from a recent study by Sanjeev et al., which reported that 31.6% of participants were housewives and 9.2% were farmers. Regarding symptomatology, 65.9% of our participants experienced itching, compared to 44.54% in a study by Chopra et al. [14] on hyperkeratotic palmoplantar eczema.

Treatment outcomes in our study were substantial. "Group A demonstrated mean percentage improvements of 60.15% ± 25.02% in the palms and 60.21% ± 23.06% in the soles. Similarly, Group B showed mean percentage improvements of 64.31% ± 27.37% in the palms and 60.48% ± 20.31% in the soles. For context, Politiek et al. found that 10 out of 23 patients with hyperkeratotic hand eczema achieved 47.6% improvement after 8-12 weeks of oral methotrexate therapy [15]. Their treatment protocol began with 5 mg weekly, progressing to a maximum of 20 mg weekly depending on clinical response.

Regarding adverse effects, cutaneous reactions were observed exclusively in Group A. These included blistering, hyperpigmentation, electric current sensations, and severe itching. One patient developed blisters after the second iontophoresis session, which resolved within seven days. Two patients experienced hyperpigmentation that cleared within one week. Two other patients reported experiencing electric shocks during treatment, while one patient developed severe itching after the initial session, which necessitated discontinuation of treatment.

In contrast, Group B primarily experienced systemic side effects, including headaches and nausea, following methotrexate administration. Headaches resolved spontaneously without intervention. Nausea occurred in four patients, with two experiencing episodes of vomiting that required treatment with ondansetron and folic acid. Laboratory abnormalities were also noted: two patients exhibited mild elevations in Serum Glutamic-Oxaloacetic Transaminase/Serum Glutamic-Pyruvic Transaminase (SGOT/SGPT) after 12 weeks of therapy, while two others developed higher levels after eight weeks, prompting cessation of methotrexate. These adverse effect profiles align with previous findings reported by Goujon et al. [16] and Weatherhead et al. [17].

To further validate our findings and refine the iontophoretic technique for enhanced clinical application, we recommend future studies with larger sample sizes and extended follow-up periods. Our current research had several limitations, including a relatively small sample size and the absence of blinding. The lack of blinding may have introduced observer bias in the evaluation of treatment outcomes, as both patients and clinicians were aware of the treatment type, potentially influencing subjective assessments of efficacy and side effects.

## Conclusions

Our study represents a unique and innovative contribution to dermatological research, as it conducts the first direct comparison between methotrexate iontophoresis and oral methotrexate for treating hyperkeratotic palmoplantar eczema. To our knowledge, no previous studies have compared these two treatment modalities specifically for palmoplantar hyperkeratotic eczema.

The findings from our investigation demonstrate that both methotrexate iontophoresis and oral methotrexate demonstrate comparable efficacy in managing hyperkeratotic palmoplantar eczema. Both therapeutic approaches resulted in similar clinical improvements, with mean percentage improvements in HECSI scores approximating 60% across the palms and soles in both treatment groups. 

Methotrexate iontophoresis was associated with significantly fewer side effects and virtually no systemic adverse reactions. The adverse effects observed with iontophoretic delivery were primarily localized and transient, including temporary blistering, hyperpigmentation, tingling sensations, and occasional itching. In contrast, patients receiving oral methotrexate frequently experienced systemic complications, particularly gastrointestinal disturbances and elevated liver enzymes. In some cases, these adverse effects necessitated complete discontinuation of therapy.

Based on these observations, iontophoretic delivery of methotrexate warrants consideration as a valuable alternative in the therapeutic armamentarium for managing palmoplantar hyperkeratotic eczema. This approach may be particularly beneficial for specific patient populations, including those with comorbidities that contraindicate systemic therapy, individuals who have previously shown intolerance to oral methotrexate, and patients who are hesitant to take oral medications. Expanding treatment options allows clinicians to better tailor therapeutic strategies to individual patient needs, potentially improving both outcomes and quality of life.
